# Autarkie und Offenheit — Überlegungen zur optimalen Balance einer offenen Volkswirtschaft

**DOI:** 10.1007/s10273-022-3271-8

**Published:** 2022-09-16

**Authors:** Thieß Petersen, Marcus Wortmann

**Affiliations:** Bertelsmann Stiftung, Carl-Bertelsmann-Straße 256, 33311 Gütersloh, Deutschland

**Keywords:** D60, F10, F15

## Abstract

Nach einem Globalisierungsschub im Zuge des Falls des Eisernen Vorhangs und von Chinas Beitritt zur WTO wurde die internationale Arbeitsteilung in den vergangenen Jahren stärker von protektionistischen Tendenzen geprägt. Die Unterbrechungen der grenzüberschreitenden Lieferkettenbeziehungen während der Coronapandemie und des Ukrainekriegs stärken zudem den Wunsch nach einer Reduzierung der Export- und insbesondere der Importabhängigkeit. Es ist zu diskutieren, wie man theoretisch zum optimalen Offenheitsgrad einer Volkswirtschaft gelangt und damit auch ein ideales Ausmaß der ökonomischen Autarkie bestimmt.

## Abstract (en)

Following a surge in globalisation in the wake of the fall of the Iron Curtain and China joining the WTO, the international division of labour has been increasingly characterised by protectionist tendencies in recent years. Negative labour market effects for individual sectors as well as the disruptions of cross-border supply chain relations during the COVID-19 pandemic and the Ukraine war have strengthened the desire in parts of the population to reduce export- and, even more so, import-dependency. This article discusses some basic theoretical considerations on the optimal degree of openness of an economy — and thus also on the ideal degree of economic autarky.

Die internationale Arbeitsteilung hat eine Reihe von positiven Auswirkungen auf die Konsummöglichkeiten für die Bevölkerung der beteiligten Länder. Zu den wichtigsten Effekten, die die Konsummöglichkeiten der Verbraucher:innen erhöhen, gehören die folgenden:

- Im Rahmen der internationalen Arbeitsteilung kann sich jede Volkswirtschaft auf die Herstellung der Produkte konzentrieren, bei denen sie Produktivitäts- und Kostenvorteile hat. So kommt es zu Spezialisierungsgewinnen, die die weltweit zur Verfügung stehende Gütermenge und damit die Konsummöglichkeiten der Menschen erhöhen.

- Die internationale Mobilität von Arbeitskräften und Kapital führt dazu, dass die weltweit zur Verfügung stehenden Produktionsfaktoren dort eingesetzt werden, wo sie den größten Beitrag zur gesamtwirtschaftlichen Wertschöpfung leisten. Für die Welt als Ganzes bedeutet dies: Es steht eine größere Menge an Gütern und Dienstleistungen zur Verfügung, für die die Verbraucher:innen einen geringeren Preis zahlen müssen.

- Die Intensivierung des Handels zwischen den Ländern erhöht den Wettbewerbsdruck und damit auch den Zwang, durch Innovationen und technischen Fortschritt die Produktionskosten zu senken, um international konkurrenzfähig zu bleiben. Eine Kostenreduzierung durch technischen Fortschritt impliziert eine Erhöhung der Produktivität, woraus sich wiederum eine Steigerung der Menge konsumierbarer Waren und Dienstleistungen ergibt.

- Wenn ein Unternehmen für den Weltmarkt produziert, bedeutet dies eine Ausweitung seiner Produktion. Häufig können damit Vorteile der Massenproduktion genutzt werden. Auch dies entspricht einer Produktivitätssteigerung.

- Die politische Einigung über eine gegenseitige Anerkennung von Produktstandards senkt die Kosten des internationalen Handels und erleichtert den grenzüberschreitenden Güteraustausch. Dies fördert das wirtschaftliche Wachstum und erhöht die zur Verfügung stehende Konsumgütermenge.

- Schließlich ist noch zu berücksichtigen, dass der Handel mit anderen Ländern den einheimischen Verbraucher:innen den Kauf von ganz neuen Produkten ermöglicht und somit die Produktvielfalt erhöht. Auch wenn dies nicht unbedingt das reale Bruttoin-

**Dr. Thieß Petersen** ist Senior Advisor der Bertelsmann Stiftung in Gütersloh im Programm „Nachhaltige Soziale Marktwirtschaft“ und Lehrbeauftragter an der Europa-Universität Viadrina in Frankfurt/Oder.

**Dr. Marcus Wortmann** ist Project Manager der Bertelsmann Stiftung in Gütersloh im Programm „Nachhaltige Soziale Marktwirtschaft“ und leitet den Themenschwerpunkt „Economics of Transformation“.

landsprodukt (BIP) steigert, bedeutet eine größere Produktvielfalt mehr Auswahlmöglichkeiten für die Konsument:innen, was als eine Verbesserung der Konsummöglichkeiten angesehen werden kann.

Die Aufnahme von internationalen Handelsaktivitäten steigert die Konsummöglichkeiten eines Landes jedoch nicht unbegrenzt. Neben der nutzenerhöhenden Ausweitung der Konsummöglichkeiten hat die internationale Arbeitsteilung auch Kosten. Die Organisation und Durchführung des grenzüberschreitenden Austauschs von Produkten, Vorleistungen und Rohstoffen nimmt produktive Ressourcen in Anspruch. Wird die Wertschöpfungskette im Kontext der Herstellung eines einfachen Konsumguts so weit ausgedehnt, dass nicht nur fünf, sondern fünfzehn Länder beteiligt sind, ergeben sich – wenn überhaupt – nur noch geringe Produktivitätszuwächse bzw. Spezialisierungsgewinne. Gleichzeitig müssen Energie, Rohstoffe und menschliche Arbeitszeit für die Durchführung der internationalen Arbeitsteilung aufgewendet werden. Zu bedenken sind zudem die Treibhausgasemissionen, die durch die internationale Arbeitsteilung entstehen, allen voran durch den grenzüberschreitenden Transport von Endprodukten, Vorleistungen und Rohstoffen. Per Saldo können diese Kosten bewirken, dass die Menge der verfügbaren Konsumgüter ab einem bestimmten Ausmaß der internationalen Arbeitsteilung sinkt.

Eine Ausweitung der internationalen Arbeitsteilung (IA) ist für ein einzelnes Land ökonomisch betrachtet also nur sinnvoll, solange der damit verbundene zusätzliche gesellschaftliche Nutzen (Grenznutzen = GN) größer ist als die zusätzlichen gesamtwirtschaftlichen Kosten (Grenzkosten = GK). Wird, wie in der volkswirtschaftlichen Analyse üblich, von steigenden Grenzkosten und sinkendem Grenznutzen ausgegangen, liegt das optimale Ausmaß der internationalen Arbeitsteilung in Abbildung 1 bei IA*.

Abbildung 1

**Gesamtwirtschaftlich optimales Ausmaß der internationalen Arbeitsteilung (IA*)**

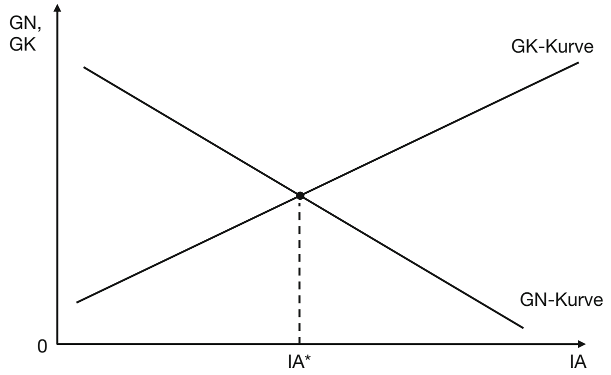

Quelle: Petersen (2020, 292).

## Konsummöglichkeitenkurve und Offenheitsgrad

Diese Überlegungen lassen sich in eine zweite Grafik übertragen. In ihr wird die Höhe des realen Konsums einer Volkswirtschaft in Abhängigkeit vom ökonomischen Offenheitsgrad des Landes abgetragen. Der Offenheitsgrad kann beispielsweise durch die Außenhandelsquote ausgedrückt werden. Sie ergibt sich, wenn die Summe aus Exporten und Importen in Relation zum BIP des Landes gesetzt wird.

Zumindest theoretisch ist es denkbar, dass ein Land sich vollständig von der internationalen Arbeitsteilung abkoppelt. Ohne den Import von Produkten, Vorleistungen und Rohstoffen aus dem Ausland verfügt das Land nur über geringe Produktions- und damit auch Konsummöglichkeiten ($C_{Autarkie}$ in Abbildung 2). Dies gilt insbesondere für rohstoffarme Länder wie Deutschland, das auf Rohstoffe aus dem Ausland angewiesen ist. Wenn sich das Land öffnet, kann es die skizzierten ökonomischen Vorteile der internationalen Arbeitsteilung nutzen. Die Konsummöglichkeiten (C) nehmen bei einem wachsenden Offenheitsgrad zu. Die Kurve C = f(OG) kann dabei als die Konsummöglichkeitenkurve bezeichnet werden.

Wenn Transport- und Organisationstätigkeiten jedoch zu viele produktive Ressourcen des Landes verbrauchen, reduziert eine weitere Ausdehnung der grenzüberschreitenden Wirtschaftsaktivitäten per saldo die Menge der zur Verfügung stehenden Konsumgüter. Es gibt somit einen Offenheitsgrad, ab dem die Konsummöglichkeiten bei einer weiteren Steigerung des Offenheitsgrads der Volkswirtschaft sinken – in Abbildung 2 ist dies bei OG* der Fall. OG* kann insofern als der optimale Offenheitsgrad angesehen werden, weil er der Bevölkerung unter sonst gleichbleibenden Rahmenbedingungen den größtmöglichen Konsum je Einwohner:in erlaubt.

Der Punkt OG* aus Abbildung 2 stimmt dabei nicht notwendigerweise mit dem Punkt IA* in Abbildung 1 überein. Grund ist, dass das jeweilige Optimum unterschiedlichen Kriterien unterliegt. Der optimale Offenheitsgrad in Abbildung 2 orientiert sich nur an den Konsummöglichkeiten der Bevölkerung. Der optimale Grad der internationalen Arbeitsteilung umfasst hingegen alle Vor- und Nachteile der internationalen Arbeitsteilung für eine Volkswirtschaft, also z. B. auch die Auswirkungen auf die Investitionen, den technologischen Fortschritt, das Ausmaß der Treibhausgasemissionen und vieles mehr.

## Lageparameter der Konsummöglichkeitenkurve

Die Konsummöglichkeitenkurve hat eine Vielzahl von Lageparametern. Dazu gehören z. B. alle Maßnahmen,

Abbildung 2

**Zusammenhang zwischen dem Offenheitsgrad (OG) und den realen Konsummöglichkeiten (C)**

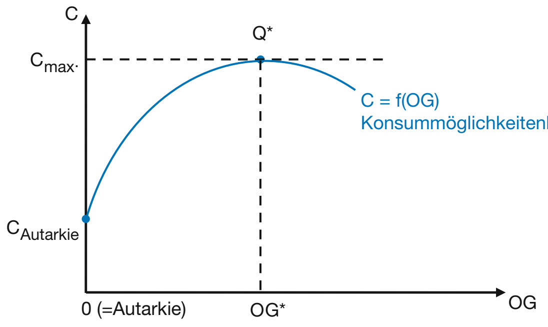

Quelle: eigene Darstellung.

die die Produktionsmöglichkeiten einer Volkswirtschaft steigern, also eine quantitative und qualitative Erhöhung des Arbeitskräfteangebots, ein Ausbau des gesamtwirtschaftlichen Kapitalstocks durch Investitionen und eine Steigerung der Produktivität durch technologischen Fortschritt. Alle diese Maßnahmen bedeuten, dass die Konsummöglichkeiten bei einem gegebenen Offenheitsgrad größer werden. Die Konsummöglichkeitenkurve wird also nach oben verschoben – inklusive des Punktes $C_{Autarkie}$.

Mit Blick auf die internationale Arbeitsteilung spielen vier weitere Aspekte eine wichtige Rolle:

- *Größe des Landes*: Je größer ein Land ist, desto größer ist der Binnenmarkt und desto weniger ist das Land auf Exporte und Importe angewiesen. Die Erhöhung des Offenheitsgrads hat daher geringere ökonomische Vorteile für das Land. Die Konsummöglichkeitenkurve einer großen Volkswirtschaft wie den USA verläuft daher flacher als bei einer kleinen Volkswirtschaft wie Belgien, Irland oder den Niederlanden.

- *Handelshemmnisse*: Der Abbau von tarifären und nichttarifären Handelshemmnissen erhöht die ökonomischen Vorteile, die sich aus Exporten und Importen sowie der damit verbundenen internationalen Arbeitsteilung ergeben. Die Konsummöglichkeitenkurve wird nach oben gedreht, der Punkt $C_{Autarkie}$ bleibt dabei konstant.

- *Risiken von Lieferkettenunterbrechungen*: Eine Unterbrechung des grenzüberschreitenden Handels, also z. B. das Ausbleiben von zu importierenden Rohstoffen und Vorleistungen im Zuge der Coronapandemie und des Ukrainekriegs, reduziert die wirtschaftlichen Vorteile des internationalen Handels. Die Konsummöglichkeitenkurve wird in diesem Fall nach unten gedreht, wobei der Punkt $C_{Autarkie}$ wiederum unverändert bleibt.

- *Einpreisung negativer externer Effekte*: Wenn zum Zwecke der Reduzierung von klimaschädlichen Treibhausgasemissionen Regierungen einen $CO_2$-Preis einführen bzw. einen bestehenden Emissionspreis erhöhen, erhöht das die Kosten der internationalen Arbeitsteilung. Die Konsumvorteile aus der internationalen Arbeitsteilung werden dadurch geringer. Erneut kommt es zu einer Drehung der Konsummöglichkeitenkurve nach unten.

## Autarkiepräferenzen der Bevölkerung

Ein Anstieg des Offenheitsgrads einer Volkswirtschaft bringt den Verbraucher:innen eines Landes die skizzierten Vorteile der internationalen Arbeitsteilung. Das bedeutet, dass ein Anstieg der Offenheit wohlfahrtserhöhende Konsequenzen hat, weil die Konsummöglichkeiten der Bevölkerung zunehmen. Allerdings ergeben sich daraus auch mindestens zwei negative Begleiterscheinungen, die die Wohlfahrt reduzieren können.

*Erstens*: Der grenzüberschreitende Austausch von Waren, Dienstleistungen, Kapital, Arbeitskräften und Technologien hat zur Folge, dass sich der Grad der Knappheit all dieser Tauschobjekte in den beteiligten Ländern verändert. Daraus ergeben sich Preisänderungen, die zu Einkommensänderungen führen. Dies lässt sich am Beispiel des globalen Arbeitsmarktes verdeutlichen. Ausgangspunkt ist die Feststellung, dass hoch entwickelte Industrienationen wie Deutschland und die USA im Vergleich zu wirtschaftlich weniger entwickelten Ländern wie China und Indien über relativ viel Kapital und relativ wenige Arbeitskräfte verfügen. In Kombination mit den unterschiedlichen Niveaus des wirtschaftlichen Wohlstands führt dies zu relativ hohen Löhnen in den Industrienationen und relativ geringen Löhnen in Schwellen- und Entwicklungsländern. Die internationale Arbeitsteilung bewirkt, dass sich diese Löhne tendenziell angleichen: Wegen des hohen Angebots an Arbeitskräften hat China einen Wettbewerbsvorteil bei arbeitsintensiv hergestellten Produkten. Die Spezialisierung auf die Herstellung und den Export dieser Güter erhöht in China die Nachfrage nach Arbeitskräften und damit die Löhne. Deutschland spezialisiert sich auf die Produktion von Gütern, für deren Herstellung viel Kapital und Technologie benötigt wird. Die Produktion arbeitsintensiv hergestellter Güter wird hingegen reduziert. Das Ergebnis ist ein Rückgang der Nachfrage nach Arbeitskräften – vor allem nach gering qualifizierten Arbeitskräften – mit einem entsprechenden Druck auf die Löhne in Deutschland. Die skizzierten Wirkungsmechanismen des internationalen Handels auf die Arbeitsmärkte in entwickelten Industrienationen sind beispielsweise von Autor et al. (2013) für die lokalen Arbeitsmärkte in den USA nachgewiesen. Die internationale Arbeitsteilung hat also für die Volkswirtschaft als Ganzes einkommenserhöhende Effekte, aber für einzelne Personengruppen und Wirtschaftsbranchen bedeutet sie auch Einkommensrückgänge.

*Zweitens*: Bei einer Intensivierung der Integration in die Weltwirtschaft ergeben sich gegenseitige Abhängigkeiten zwischen den Volkswirtschaften. Diese können deutliche Risiken bergen: So müssten die exportierenden einheimischen Unternehmen ihre Produktion – und mit ihr das Beschäftigungsniveau im Inland – verringern, wenn ihre Exporte wegen einer Rezession im Ausland zurückgehen. Noch gravierender können Importabhängigkeiten sein. Preiserhöhungen bei wichtigen Importgütern können z. B. den Inflationsdruck erhöhen. Wenn Vorleistungen oder Rohstoffe aus dem Ausland nicht oder nur noch unzureichend geliefert werden, kann das zu Produktionsunterbrechungen und Versorgungsengpässen im Inland führen. Gleiches gilt, wenn das Inland bestimmte Produkte gar nicht mehr selbst herstellt, sondern die einheimische Nachfrage komplett durch Importe deckt. Als besonders kritisch kann sich eine starke einseitige Abhängigkeit von wichtigen Ressourcen oder Energieträgern herausstellen, wenn ganze Wertschöpfungsketten ohne die stete Zufuhr von außen nicht mehr funktionieren oder gar die heimische Energieversorgung gefährdet ist. Neben der Angewiesenheit auf fossile Energieträgerimporte, etwa aus Russland, sind für Deutschland zahlreiche weitere Rohstoffabhängigkeiten, unter anderem aus China, als kritisch zu bewerten, die auch für das Gelingen zentraler Entwicklungsziele wie das der Energiewende maßgeblich sind (Fremerey und Obst, 2022).

Solche kritischen Abhängigkeiten beschränken sich allerdings nicht nur auf die Ressourcen- und Energiezufuhr, den Güterhandel und die kurzfristige Produktionsfähigkeit und Versorgungslage einer Volkswirtschaft. Auch in technologischer Hinsicht kann gerade bei steigendem Innovationstempo die Gefahr bestehen, vom Ausland und den dortigen Standards und Entwicklungen abhängig zu werden. Verliert die heimische Volkswirtschaft an Innovationskraft und kann der technologische Fortschritt mit internationalen Wettbewerber:innen nicht mehr Schritt halten, wäre die Wettbewerbsfähigkeit der inländischen Unternehmen bedroht. Dies könnte sich wiederum nicht nur unmittelbar negativ auf Exporte, Wertschöpfung und Beschäftigung im Inland auswirken, sondern auch die langfristigen wirtschaftlichen Entwicklungsperspektiven sowie die nationale Gestaltungsmacht bei neuen Technologien beeinträchtigen. Tatsächlich fordert insbesondere China bei vielen Zukunftstechnologien die Führungsposition westlicher Staaten heraus (Breitinger et al., 2020). Eine gewisse Technologiesouveränität sollte deshalb gewahrt bleiben (Edler et al., 2020).

Viele weitere (insbesondere politische und geostrategische) Risiken können mit einer ausgeprägten und zu wenig diversifizierten Außenhandelsverflechtung verbunden sein, wenn zu große Abhängigkeiten in politische Erpressbarkeit oder Einflussnahme münden. Diese problematischen Auswirkungen einer völlig offenen Volkswirtschaft haben zur Folge, dass es in einer Volkswirtschaft auch eine Präferenz für eine Reduzierung der internationalen Arbeitsteilung bzw. ein mehr oder weniger stark ausgeprägtes Autarkiestreben gibt.

## Konsum-Offenheitsgrad-Indifferenzkurve

Die Frage, welches Ausmaß der Offenheit aus gesamtgesellschaftlicher Sicht am besten ist, lässt sich nicht eindeutig beantworten. Auf den ersten Blick könnte vermutet werden, dass der Offenheitsgrad OG* in Abbildung 2 optimal ist, weil sich damit die größten Konsummöglichkeiten erzielen lassen. Diese Einschätzung ist jedoch nur zutreffend, wenn sich die Gesellschaft ausschließlich am Ziel möglichst hoher Konsummöglichkeiten orientiert. Zwingend notwendig ist diese Zielsetzung jedoch nicht.

Es ist ebenso gut möglich, dass die Gesellschaft als Ganzes eine Präferenz für eine gewisse Autarkie hat und damit eine Aversion gegen einen zu hohen Offenheitsgrad. Diese Aversion resultiert aus den genannten negativen Begleiterscheinungen einer Einbindung in die internationale Arbeitsteilung – also den Export- und Importabhängigkeiten sowie den negativen Einkommenseffekten für bestimmte Personengruppen bzw. Sektoren. Um den individuellen Nutzen einer Person zu verringern, muss es noch nicht einmal zu einer Einkommensreduzierung kommen. Schon die Befürchtung, den eigenen Arbeitsplatz wegen der Konkurrenz durch ausländische Anbieter:innen zu verlieren, kann zu einer individuellen Nutzeneinbuße führen – und damit auch zu einer gesamtgesellschaftlichen Wohlfahrtseinbuße.

Die mit diesen nutzen- bzw. wohlfahrtsreduzierenden Effekten der internationalen Arbeitsteilung verbundene Autarkiepräferenz äußert sich in einer entsprechenden Wohlfahrtsfunktion. Sie ordnet jeder Kombination aus Konsummöglichkeiten und ökonomischem Offenheitsgrad der Volkswirtschaft einen bestimmten Wert des gesellschaftlichen Nutzens zu. Die Höhe der Wohlfahrt hängt in positiver Weise von der Höhe der Konsummöglichkeiten und in negativer Weise vom Ausmaß der negativen Effekte der ökonomischen Offenheit ab. Dies bedeutet: Eine Steigerung der Konsummöglichkeiten erhöht die Wohlfahrt der Gesellschaft, während eine Erhöhung des Offenheitsgrads die Wohlfahrt verringert. Alle Kom-

Abbildung 3

**Gesamtgesellschaftliche Indifferenzkurve einer Volkswirtschaft**

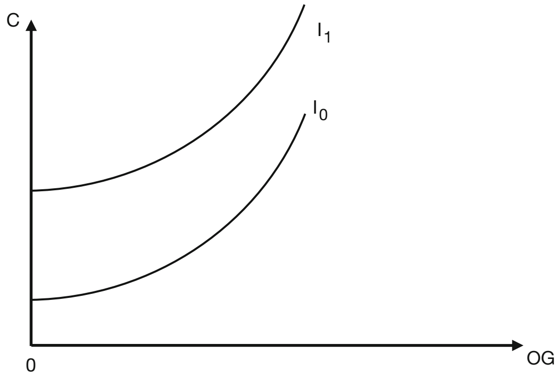

Quelle: eigene Darstellung.

binationen aus Konsummöglichkeiten und ökonomischer Offenheit, die der Gesellschaft denselben Nutzen stiften, liegen auf einer sozialen Indifferenzkurve (I).

Im Fall einer Autarkiepräferenz – und damit einer Offenheitsaversion – bleibt der gesellschaftliche Nutzen bei einem Anstieg des Offenheitsgrads nur dann unverändert, wenn gleichzeitig die Konsummöglichkeiten steigen. Der daraus resultierende Verlauf der sozialen Indifferenzkurve ist in Abbildung 3 dargestellt. Dabei gilt zudem: Je weiter eine soziale Indifferenzkurve vom Ursprung entfernt ist, desto größer ist der gesellschaftliche Nutzen bzw. die gesellschaftliche Wohlfahrt (W), d. h. es gilt: $W(I_1) > W(I_0)$.

Die Lage dieser Indifferenzkurve hängt wiederum von verschiedenen Einflussfaktoren ab. Zwei von ihnen sind:

- *Ausmaß der Unsicherheitsaversion*: Wie weiter oben beschrieben, ist die Einbindung einer Volkswirtschaft in die internationale Arbeitsteilung mit Unsicherheiten verbunden. Das betrifft die ökonomische Abhängigkeit vom Ausland – sowohl als Abnehmer:innen der heimischen Waren und Dienstleistungen als auch als Liefernde von Rohstoffen, Vorleistungen und Endprodukten – und die veränderten Knappheiten im Inland. Letzteres kann für einzelne Personengruppen oder Branchen negative Auswirkungen auf das Produktions- und Beschäftigungsvolumen haben und damit auch auf die Einkommenshöhe. Eine Unsicherheit ergibt sich daraus, dass nicht vorhersehbar ist, ob es zu diesen negativen Entwicklungen kommt bzw. welche Personen davon betroffen sein werden. Je höher die Unsicherheitsaversion in einer Gesellschaft ausgeprägt ist, desto größer muss im Fall einer Erhöhung des ökonomischen Offenheitsgrads der wohlfahrtskompensierende Zuwachs an Konsummöglichkeiten sein. Die Indifferenzkurve hat daher einen steileren Verlauf.

- *Ausmaß der sozialen Absicherung durch den Staat*: Zur Abfederung von negativen Auswirkungen, die sich aus der internationalen Arbeitsteilung ergeben können, bieten sich staatliche Kompensationsmaßnahmen an. Das betrifft vor allem die sozialen Sicherungssysteme, die die privaten Haushalte finanziell unterstützen, wenn es dort zu globalisierungsbedingten Einkommensrückgängen kommt. Je stärker dieses Sicherheitsnetz ausgeprägt ist, desto geringer ist die Gefahr, dass die verfügbaren realen Einkommen wegen der Einbindung in die internationale Arbeitsteilung reduziert werden. Das erhöht die Akzeptanz der Globalisierung. Die Indifferenzkurve hat – im Vergleich zu einer Situation ohne staatliche Sicherungssysteme – einen flacheren Verlauf, weil der Zuwachs an Konsummöglichkeiten weniger groß sein muss als ohne diesen Schutz.

## Optimaler Grad der ökonomischen Offenheit

Um den wohlfahrtsmaximierenden ökonomischen Offenheitsgrad grafisch zu bestimmen, muss die Konsummöglichkeitenkurve mit der sozialen Indifferenzkurve zusammengebracht werden. Die aus Sicht der Gesellschaft optimale Kombination aus Konsummöglichkeiten und ökonomischer Offenheit liegt dort, wo die soziale Indifferenzkurve die Konsummöglichkeitenkurve tangiert. In Abbildung 4 ist dies im Punkt $Q^{opt.}$ der Fall. Bei einer gesamtgesellschaftlichen Autarkiepräferenz ist der wohlfahrtsmaximierende Offenheitsgrad der Volkswirtschaft also geringer als der Offenheitsgrad, der die maximalen Konsummöglichkeiten erlaubt (OG*).

## Veränderung des optimalen Grads

Der optimale Offenheitsgrad einer Volkswirtschaft verändert sich, wenn sich die Lage bzw. der Verlauf der beiden involvierten Kurven verändert.

Die Lage der Konsummöglichkeitenkurve verändert sich, wie weiter oben beschrieben, unter anderem durch die Einführung zusätzlicher Handelshemmnisse. Sie reduzieren den gesamtwirtschaftlichen Vorteil, der sich aus der internationalen Arbeitsteilung für ein Land ergibt. Die Konsummöglichkeitenkurve wird nach unten gedreht, wobei sich der Punkt mit den Konsummöglichkeiten im Fall der Autarkie ($C_{Autarkie}$) nicht verändert. Die Drehung der Konsummöglichkeitenkurve nach unten hat zur Folge, dass der Offenheitsgrad, der zum größtmöglichen realen Konsum führt, geringer wird ($OG_1^* < OG_0^*$, vgl. Abbildung 5).

Abbildung 4
**Grafische Bestimmung des optimalen Offenheitsgrads einer Volkswirtschaft**

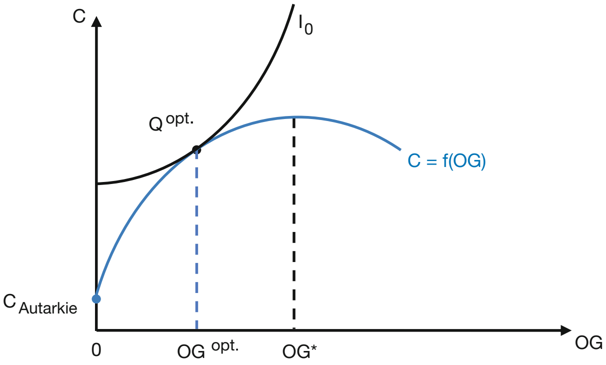

Quelle: eigene Darstellung.

Abbildung 5
**Veränderung des optimalen Offenheitsgrads bei Einführung zusätzlicher Handelshemmnisse**

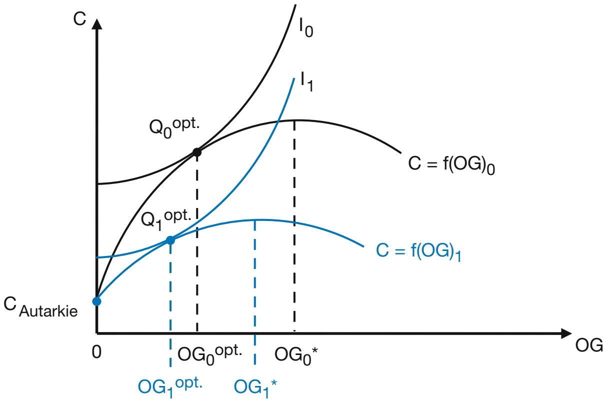

Quelle: eigene Darstellung.

Dieses Ergebnis passt zur Bestimmung des optimalen Ausmaßes der internationalen Arbeitsteilung in Abbildung 1: Handelshemmnisse reduzieren die Vorteile, die ein Land aus der internationalen Arbeitsteilung ziehen kann. Für jedes gegebene Ausmaß der internationalen Arbeitsteilung (IA in Abbildung 1) ergibt sich daher ein geringerer gesamtgesellschaftlicher Grenznutzen der internationalen Arbeitsteilung. Die Grenznutzenkurve wird in Abbildung 1 nach unten verschoben. Die Folge ist eine Verringerung des optimalen Ausmaßes der internationalen Arbeitsteilung (IA*) für das Land. Der gleiche Effekt ergibt sich, wenn der Transport von Waren mit fossiler Energie erfolgt und die mit dem Energieverbrauch verbundenen Treibhausgasemissionen mit einer $CO_2$-Steuer (oder einem Preis für Emissionszertifikate) belegt werden. Alternativ kann in diesen beiden Fällen die Grenznutzenkurve unverändert bleiben und dafür die Grenzkostenkurve nach oben verschoben werden, was ebenfalls zu einer Verringerung des optimalen Ausmaßes der internationalen Arbeitsteilung führt.

Ob damit auch der optimale – also der wohlfahrtsmaximierende – Offenheitsgrad der Volkswirtschaft geringer wird, hängt vom Verlauf der gesamtgesellschaftlichen Indifferenzkurve ab. In Abbildung 5 reduziert sich der optimale Offenheitsgrad. Zwingend notwendig ist dies jedoch nicht. Zumindest theoretisch ist es auch denkbar, dass der aus gesamtgesellschaftlicher Sicht optimale Offenheitsgrad größer wird. Das wäre z. B. der Fall, wenn die Gesellschaft eine hohe Konsumpräferenz hat. In diesem Fall bewirken Handelsbeschränkungen oder höhere Transportkosten als Folge eines höheren $CO_2$-Preises eine Reduktion der gesamtgesellschaftlichen Konsummöglichkeiten. Um diese Konsumeinschränkung zu kompensieren, kann die Gesellschaft ihren Außenhandel intensivieren, um höhere globalisierungsbedingte Spezialisierungsgewinne zu realisieren, die dann die Konsummöglichkeiten der Bevölkerung erhöhen.

Neben der Konsummöglichkeitenkurve kann sich auch die Lage bzw. der Verlauf der gesamtgesellschaftlichen Indifferenzkurve verändern. Externe Schocks, die den Import von essenziellen Vorleistungen und Konsumprodukten temporär verhindern, können in weiten Teilen der Bevölkerung den Wunsch nach einer höheren Autarkie stärken. So wurden beispielsweise zu Beginn der Coronapandemie in der gesellschaftspolitischen Debatte in Deutschland verstärkt Forderungen laut, die Abhängigkeit vom Import besonders wichtiger Produkte – also z. B. Medikamente, medizinische Geräte und andere lebenswichtige Produkte – zu verringern (Flach und Steininger, 2020, 17). Und der Ausbruch des Ukrainekriegs führt zu einem größeren Wunsch nach einer deutschen Energieautarkie (Grimm et al., 2022, 254).

Wenn somit die Aversion gegen eine ökonomische Offenheit zunimmt, verläuft die gesamtwirtschaftliche Indifferenzkurve steiler: Um trotz einer wachsenden Offenheit das identische Wohlfahrtsniveau zu erreichen, ist bei einer höheren Autarkiepräferenz ein stärkerer Anstieg der Konsummöglichkeiten erforderlich. Damit verändert sich auch der Tangentialpunkt, der den optimalen Offenheitsgrad festlegt. Bei einer steiler verlaufenden Indifferenzkurve liegt dieser Punkt links von $Q^{opt.}$ in Abbildung 4. Der wohlfahrtsmaximierende Offenheitsgrad wird somit geringer.

## Ein neues außenwirtschaftliches Gleichgewicht

Die bisherigen Überlegungen verdeutlichen das Spannungsverhältnis zwischen den Vor- und Nachteilen der internationalen Arbeitsteilung, also in erster Linie dem Nutzen aus höheren Konsummöglichkeiten einerseits und den damit einhergehenden Risiken aus außenwirtschaftlichen Abhängigkeiten anderseits. Beide Faktoren entwickeln sich derzeit eher gegen die Vorteilhaftigkeit einer zumindest uneingeschränkten Offenheit. Während sich die jahrzehntelang vorherrschenden handels- und wachstumsfreundlichen globalen Rahmenbedingungen nun deutlich verschlechtern, wachsen bedingt durch den Klimawandel, die Coronapandemie und den Krieg gegen die Ukraine nun die Bestrebungen, künftig aus politisch strategischen Gründen unabhängiger und resilienter zu werden (Bertelsmann Stiftung, 2022). Das in Deutschland gültige Staatsziel eines außenwirtschaftlichen Gleichgewichts, welches seit langer Zeit durch ohnehin extreme Außenhandelsüberschüsse infrage stand, bedarf vor diesem Hintergrund eines neuen Verständnisses.

Ein zeitgemäßes außenwirtschaftliches Gleichgewicht kann sich dabei nicht mehr allein auf den Saldo von Ex- und Importen bzw. den damit verbundenen Kapitalströmen beziehen, sondern muss entlang der hier skizzierten und auf den ersten Blick widerstrebenden Ziele der Autarkie und Offenheit gefunden werden. Der optimale Grad der Offenheit – größtmögliche Handelsvorteile bei geringen (kritischen) Abhängigkeiten – muss allerdings nicht notwendigerweise mit einem geringeren Handelsvolumen einhergehen, sondern kann auch durch eine stärkere Diversifizierung bestehender Handelsbeziehungen erreicht werden. Welche Handelsbeziehung dies genau betrifft, welche Güter, Rohstoffe, Technologien und Länder überhaupt als kritisch zu beurteilen sind, bedarf dringend umfangreicher politischer Debatten und wissenschaftlicher Bewertungsmaßstäbe. Auch die wirtschaftspolitischen Steuerungsmöglichkeiten und Instrumente müssen entsprechend dieser veränderten Zielsetzung angepasst werden. Natürlich müssen internationale Handelsbeziehungen derweil das Ergebnis betriebswirtschaftlicher Entscheidungen bleiben. Doch stärker als früher sollte der Staat ein Auge darauf haben, wie stark sich die Volkswirtschaft insgesamt von den Entwicklungen andernorts abhängig macht und ob diese Abhängigkeiten eigentlich den gesellschaftlichen Autarkiepräferenzen entsprechen. Dafür gibt es zahlreiche wirtschaftspolitische Stellhebel, die Autarkie und Resilienz erhöhen können, ohne dabei Außenhandelspotenziale per se zu beschränken. Besonders aus Sicht ressourcenarmer Länder wie Deutschland kann etwa die Importabhängigkeit bei Rohstoffen und Energieträgern erheblich reduziert werden, wenn es gelingt, eine stärkere Kreislaufführung zu etablieren, die erneuerbaren Energien auszubauen sowie Effizienz- und Suffizienzpotenziale zu heben.
